# Prevalence of Behavioral Flags in the Electronic Health Record Among Black and White Patients Visiting the Emergency Department

**DOI:** 10.1001/jamanetworkopen.2022.51734

**Published:** 2023-01-19

**Authors:** Anish K. Agarwal, Emily Seeburger, Gerald O’Neill, Chidinma C. Nwakanma, Lillian E. Marsh, Kevin Alexander Soltany, Eugenia C. South, Ari B. Friedman

**Affiliations:** 1 Department of Emergency Medicine, University of Pennsylvania, Perelman School of Medicine, Philadelphia; 2 Center for Emergency Care Policy and Research, Philadelphia, Pennsylvania; 3 Penn Medicine Center for Health Care Innovation, Philadelphia, Pennsylvania; 4 Wake Forest School of Medicine, Winston-Salem, North Carolina

## Abstract

**Importance:**

Behavioral flags in the electronic health record (EHR) are designed to alert clinicians of potentially unsafe or aggressive patients. These flags may introduce bias, and understanding how they are used is important to ensure equitable care.

**Objective:**

To investigate the incidence of behavioral flags and assess whether there were differences between Black and White patients and whether the flags were associated with differences in emergency department (ED) clinical care.

**Design, Setting, and Participants:**

This was a retrospective cohort study of EHR data of adult patients (aged ≥18 years) from 3 Philadelphia, Pennsylvania, EDs within a single health system between January 1, 2017, and December 31, 2019. Secondary analyses excluded patients with sickle cell disease and high ED care utilization. Data were analyzed from February 1 to April 4, 2022.

**Main Outcomes and Measures:**

The primary outcome of interest was the presence of an EHR behavioral flag. Secondary measures included variation of flags across sex, race, age, insurance status, triage status, ED clinical care metrics (eg, laboratory, medication, and radiology orders), ED disposition (discharge, admission, or observation), and length of key intervals during ED care.

**Results:**

Participating EDs had 195 601 eligible patients (110 890 [56.7%] female patients; 113 638 Black patients [58.1%]; 81 963 White patients [41.9%]; median [IQR] age, 42 [28-60] years), with 426 858 ED visits. Among these, 683 patients (0.3%) had a behavioral flag notification in the EHR (3.5 flags per 1000 patients), and it was present for 6851 ED visits (16 flagged visits per 1000 visits). Patient differences between those with a flag and those without included male sex (56.1% vs 43.3%), Black race (71.2% vs 56.7%), and insurance status, particularly Medicaid insurance (74.5% vs 36.3%). Flag use varied across sites. Black patients received flags at a rate of 4.0 per 1000 patients, and White patients received flags at a rate of 2.4 per 1000 patients (*P* < .001). Among patients with a flag, Black patients, compared with White patients, had longer waiting times to be placed in a room (median [IQR] time, 28.0 [10.5-89.4] minutes vs 18.2 [7.2-75.1] minutes; *P* < .001), longer waiting times to see a clinician (median [IQR] time, 42.1 [18.8-105.5] minutes vs 33.3 [15.3-84.5] minutes; *P* < .001), and shorter lengths of stay (median [IQR] time, 274 [135-471] minutes vs 305 [154-491] minutes; *P* = .01). Black patients with a flag underwent fewer laboratory (eg, 2449 Black patients with 0 orders [43.4%] vs 441 White patients with 0 orders [36.7%]; *P* < .001) and imaging (eg, 3541 Black patients with no imaging [62.7%] vs 675 White patients with no imaging [56.2%]; *P* < .001) tests compared with White patients with a flag.

**Conclusions and Relevance:**

This cohort study found significant differences in ED clinical care metrics, including that flagged patients had longer wait times and were less likely to undergo laboratory testing and imaging, which was amplified in Black patients.

## Key Points

**Question:**

How often are behavioral flags used in the emergency department (ED), who receives them, and is the presence of flags associated with clinical care?

**Findings:**

In this cohort study of 426 858 ED visits among 195 691 patients, 683 patients had a behavioral flag (3.5 flags per 1000 patients), with significant differences in presence of a flag noted across age, sex, insurance status, and race. Black patients were flagged at higher rates than White patients (4.0 flags vs 2.4 flags per 1000 patients).

**Meaning:**

This cohort study found that behavioral flags were infrequently used in the ED, but when they were used, disparities by several patient-level characteristics were noted.

## Teaser

This cohort study investigates the incidence of behavioral flags in electronic health records (EHR) of patients seeking care in the emergency department (ED), whether there were patient-level differences in how the flags were used, and whether the presence of a flag was associated with clinical care.

## Introduction

The actions of Black individuals in the United States have been legally criminalized for hundreds of years through Slave Codes, Black Codes, and then Jim Crow laws. After the passage of the 1964 Civil Rights Act, the criminalization of Black individuals has continued in subtle, seemingly race-neutral policies, such as Stop and Frisk policing.^1^ Medicine is not immune from the racist tropes, policies, and structures that have allowed a range of normal behaviors to be criminalized among Black individuals. The literature around the treatment of acute pain, often within the emergency department (ED), is instructive. Black adult and pediatric patients with acutely painful conditions, such a long-bone fractures, appendicitis, or ureteral colic, receive less pain management compared with White patients with similar presentations.^2,3^ One reason that has been explored in the literature is that health care practitioners may have a mindset of assumed criminality toward Black patients that influences care, including the provision of pain medications.^4^ This so-called *shadow diagnosis* is particularly prevalent for psychiatric or behavioral diagnoses and presentations.

Behavior in clinical settings is increasingly being formally documented in the electronic health record (EHR). Behavioral flags or notifications have been implemented with the purpose of mitigating violence against health care workers, a problem that has been increasing rapidly over the last several years.^5^ Approximately three-quarters of ED nurses and one-quarter of ED physicians have experienced physical assault within their career, and verbal assault is nearly ubiquitous.^5^ In the absence of evidence-based interventions to reduce staff-experienced violence, behavioral flags placed by staff after incidents of behavior deemed to be threatening or disruptive serve as an early notification of safety concerns when caring for these patients in future encounters. In addition to their intended role as notifications to staff to take precautions to potentially reduce harm from violent encounters, behavioral flags may also serve as a type of diagnosis, functioning as a biased third actor in the examination room (in addition to the patient and clinician). However, little is known about how many patients have a flag in their EHR, the demographic characteristics of who gets a flag, and whether the presence of a flag is associated with patient care. This lack of evidence on efficacy and fairness raises the possibility that the processes, people, and systems that cause behavioral flags to be placed may worsen existing societal and health care racial biases.

The objectives of this study were to investigate EHR data on ED encounters across a large urban academic health system to quantify the presence of behavioral flags and assess whether differences exist between Black and White patients and in regards to ED clinical care. We hypothesized in advance of data collection that the use of EHR behavioral flags would be small relative to the number of ED visits but that there would be variation in the use of these flags by patient race.

## Methods

### Study Design

We conducted a retrospective cohort study of EHRs for patients at 3 urban EDs. This study was approved by the University of Pennsylvania institutional review board, and the requirement for informed consent was waived given the data were de-identified. This study follows the Strengthening the Reporting of Observational Studies in Epidemiology (STROBE) reporting guideline for observational studies.

### Data Source and Population

We used data from University of Pennsylvania Health System (UPHS) EHR via the Epic Clarity database, obtained through the Penn Data Analytics Center. Inclusion criteria were patient visits at a Philadelphia, Pennsylvania–based ED within UPHS (Hospital of the University of Pennsylvania [HUP], Penn Presbyterian Medical Center [PPMC], and Pennsylvania Hospital [PAH]) between January 1, 2017, and December 31, 2019. The HUP and PAH EDs are academic EDs; HUP receives approximately 62 000 ED visits per year, and PAH receives approximately 43 000 ED visits per year. PPMC is an academic, level 1 trauma center, with approximately 48 000 ED visits per year.

Our primary aim was to investigate the presence of an EHR behavioral flag for an ED encounter and to compare the presence of flags between Black and White patients. We included visits from 2017 to 2019, regardless of whether the patient ever had a behavioral flag placed. Patients whose EHR contained a behavioral flag were then coded to indicate the flag’s presence. At UPHS, all clinical practitioners, including nurses, technicians, physicians, and resident physicians, can place a behavioral flag within a patient’s EHR.

Patients whose EHR indicated that they were White or Hispanic White were grouped as White, and those whose EHR indicated that they were Black or Hispanic Black were grouped as Black. Patient race and ethnicity data are self-reported and entered in the EHR. Approximately 95% of all UPHS ED visits were from non-Hispanic Black or non-Hispanic White patients. Hispanic White and Hispanic Black patients were grouped in with their respective non-Hispanic populations as a way to include their experiences without having the statistical power to separately analyze them. Visits from patients of other races and ethnicities were excluded from our analysis due to small sample size.

Patients who have frequent utilization of the ED, defined as having more than 18 visits (a yearly mean of >6 visits per calendar year, consistent with prior studies^6,7^) during the study period were separately analyzed. Patient with frequent ED utilization are more likely to have complex behavioral health needs, have a greater number of comorbidities, and have more psychiatric admissions; thus, they were excluded for being nonrepresentative of the rest of the patient population. Patients whose chief concern was related to sickle cell disease or sickle cell pain crisis also were analyzed separately, since sickle cell disease disproportionately impacts Black patients, and the behavior of those experiencing a pain crisis may be more likely to be interpreted by clinicians as meriting a flag.^16^

### Measures

The primary outcome measure was presence of a behavioral flag within the EHR. Patient-level variables included race, sex, age, and primary payer information. ED visit–level information studied included emergency severity index (ESI) level, hospital site, disposition from the ED (eg, discharge, admission, observation, left without seeing a clinician), waiting and length of visit times, and number and category of imaging, laboratory, and medication orders.

### Statistical Analysis

Total numbers of patients and visits were computed overall and stratified by behavioral flag presence and patient race. Proportions of Black patients and White patients with behavioral flags were compared using a binomial 2-sample test of proportions. The distribution of categorical variables between patients with and without a flag were compared by χ^2^ tests. Group comparisons of ordinal data were assessed using the nonparametric Mann-Whitney *U* test. We included a sensitivity analysis by excluding Hispanic patients regardless of race, which did not produce any substantively different findings. We also conducted a Poisson regression model analysis that estimated the incidence of a behavioral flag in a patient’s EHR with adjustment for patient and department covariates. Regressions included month and year fixed effects to account for potential temporal trends and seasonality. Standard errors were clustered by month to account for potential autocorrelation. Analyses were conducted in Stata statistical software version 17.0 (StataCorp). Results with a 2-tailed *P* < .05 were considered statistically significant. Data were analyzed from February 1 to April 4, 2022.

## Results

From 2017 to 2019, there were 426 858 ED visits across 3 EDs among 195 601 patients (110 890 [56.7%] female patients; 113 638 Black patients [58.1%]; 81 963 White patients [41.9%]; median [IQR] age, 42 [28-60] years). A total of 683 patients (0.3%) had a behavioral flag notification in the EHR (3.5 flags per 1000 patients), and a flag was present for 6851 ED visits (16 flagged visits per 1000 visits). Significant patient differences between those with a flag and those without included male sex (56.1% vs 43.3%), Black race (71.2% vs 56.7%), and insurance status, particularly Medicaid insurance (74.5% vs 36.3%). Flag use varied across sites (Table 1).

**Table 1.** Patient- and Visit-Level Characteristics Comparing All Patients With and Without Behavioral Flags Across 3 ED Sites

Characteristic	Patients, No. (%) (N = 195 601)		*P* value
	Flag (n = 683)	No flag (n = 194 918)	
Total visits, No.	6851	420 007	NA
Age, y			
18-24	70 (10.3)	31 427 (16.1)	<.001
25-34	163 (23.9)	43 414 (22.3)	
35-44	172 (25.2)	28 225 (14.5)	
45-54	137 (20.1)	26 577 (13.6)	
55-64	106 (15.5)	28 712 (14.7)	
≥65	35 (5.1)	36 563 (18.8)	
Sex			
Female	300 (43.9)	110 590 (56.7)	<.001
Male	383 (56.1)	84 327 (43.3)	
Race^a^			
Black	486 (71.2)	113 152 (58.1)	<.001
White	197 (28.8)	81 766 (42.0)	
Insurance status			
Commercial	437 (6.4)	42 065 (10.2)	<.001
Managed care	223 (3.3)	90 254 (21.9)	
Medicaid	5101 (74.5)	149 817 (36.3)	
Medicare	964 (14.1)	107 948 (26.1)	
Self-pay	126 (1.8)	23 064 (5.6)	
Emergency Severity Index level			
1 (most acute)	11 (0.2)	2615 (0.7)	<.001
2	1619 (24.4)	92 566 (23.0)	
3	3128 (47.2)	209 564 (52.1)	
4	1119 (16.7)	83 318 (20.7)	
5 (least acute)	646 (9.8)	12 007 (3.0)	
Psychiatric	106 (1.6)	2339 (0.6)	
ED site			
1 (tertiary referral center)	3294 (48.1)	168 153 (40.7)	<.001
2	2085 (30.4)	109 924 (26.6)	
3 (level 1 trauma center)	1472 (21.5)	135 079 (32.7)	

Abbreviations: ED, emergency department; NA, not applicable.

^a^ Individuals reporting Hispanic/Latinx ethnicity were included within Black and White race categories.

Compared with visits without a behavioral flag, during visits in which a flag was present, patients were evaluated in the ED for less time overall (median [IQR] time, 292 [173-466] minutes vs 284 [149-461] minutes; *P* = .01). Flagged patient ED encounters were more likely to end in a patient leaving against medical advice or without being seen by a clinician and less likely to be admitted, placed in observation, or discharged (eTable 1 in Supplement 1). Significant differences were observed in diagnostic workup orders between groups, with patients with flags more likely to have no or fewer laboratory tests ordered. Patients with flags were also less likely to have imaging tests ordered and had fewer medications ordered compared with patients with no behavioral flag (eTable 1 in Supplement 1).

Table 2 compares visit-level variables by race among 6851 patient visits in which a flag was in place. Black patients were flagged at a rate of 4.0 flags per 1000 patients, and White patients were flagged at a rate of 2.4 flags per 1000 patients (*P* < .001). Black patients with a flag, compared with White patients with a flag, had longer waiting room times (median [IQR] time, 28.0 [10.5-89.4] minutes vs 18.2 [7.2-75.1] minutes; *P* < .001) and time to see a clinician (median [IQR] time, 42.1 [18.8-105.5] minutes vs 33.3 [15.3-84.5] minutes; *P* < .001), but a shorter in-room time and total length of stay. Black patients with flags had more visits with no laboratory orders (2449 Black patients with 0 orders [43.4%] vs 441 White patients with 0 orders [36.7%]; *P* < .001) or imaging orders (3541 Black patients with no imaging [62.7%] vs 675 White patients with no imaging [56.2%]; *P* < .001) compared with White patients with a flag. When including all patient visits, Black patients with a flag were more often admitted (844 patients [14.9%]) compared with White patients with a flag (145 patients [12.1%]). When removing sickle cell–related chief concerns, Black patients with a flag were more likely to be discharged or leave against medical advice or prior to completion of treatment compared with White patients with a flag (eTable 2 in Supplement 1). Differences also existed between Black and White patients across ED site and insurance status. Black patients were more likely to be assigned an ESI level 4 (ie, less acute) compared with White patients.

**Table 2.** Visit-Level Characteristics Comparing Black and White Patients With Behavioral Flags Across 3 EDs

	Visits, No. (%) (N = 6851)		*P* value
	Black patients (n = 5649)	White patients (n = 1202)	
Total patients with a flag (No. per 1000 patients)	456 (4.0)	197 (2.4)	<.001
Visit times, median (IQR), min			
Waiting room time	28.0 (10.5-89.4)	18.2 (7.2-75.1)	<.001
Wait to see clinician	42.1 (18.8-105.5)	33.3 (15.3-84.5)	<.001
In-room time	220.1 (103.0-395.5)	246.1 (123.7-425.0)	.001
Length of stay	274 (135-471)	305 (154-491)	.01
Laboratory testing orders, No.			
0	2449 (43.4)	441 (36.7)	<.001
1-3	685 (12.1)	159 (13.2)	
≥4	2515 (44.5)	602 (50.1)	
Medication orders			
0	1463 (25.9)	351 (29.2)	.001
1-3	1910 (33.8)	436 (36.3)	
≥4	2276 (40.3)	415 (34.5)	
Radiology orders			
None	3541 (62.7)	675 (56.2)	<.001
Radiography only	1365 (24.2)	265 (22.1)	
Advanced imaging^a^	743 (13.2)	262 (21.8)	
Disposition			
Admit	844 (14.9)	145 (12.1)	.005
Discharge	3723 (65.9)	808 (67.2)	
Left against medical advice or left without seeing a clinician	855 (15.1)	192 (16.0)	
Observation	91 (1.6)	32 (2.7)	
Transfer	46 (0.8)	15 (1.3)	
Triaged	20 (0.4)	2 (0.2)	
Voided or other	70 (1.2)	8 (0.7)	
ED site			
1 (tertiary referral center)	2794 (49.5)	500 (41.6)	<.001
2	1569 (27.8)	516 (42.9)	
3 (level 1 trauma center)	1286 (22.8)	186 (15.5)	
Insurance status			
Commercial	394 (7.0)	43 (3.6)	<.001
Managed care	152 (2.7)	71 (5.9)	
Medicaid	4417 (78.2)	684 (56.9)	
Medicare	586 (10.4)	378 (31.5)	
Self-pay	100 (1.8)	26 (2.2)	
Emergency Severity Index level			
1 (most acute)	7 (0.1)	4 (0.3)	<.001
2	1282 (22.7)	337 (28.0)	
3	2568 (45.5)	560 (46.6)	
4	957 (16.9)	162 (13.5)	
5 (least acute)	558 (9.9)	88 (7.3)	
Psychiatric	87 (1.5)	19 (1.6)	
Unknown	190 (3.4)	32 (2.7)	

Abbreviation: ED, emergency department.

^a^ Including computed tomography, magnetic resonance imaging, and ultrasonography.

A Poisson regression model analysis revealed similar results (Table 3). In this model, Black patients had a statistically significant incidence rate ratio (IRR) of having an EHR flag present (IRR, 2.52; 95% CI 2.31-2.76) compared with White patients. Other significant variable associated with prevalence for an EHR flag in this model included age with strong association for ages 35 to 44 years (IRR, 2.54; 95% CI, 2.16-3.00), male sex (IRR, 2.41; 95% CI, 2.19-2.65), arrival by ambulance (IRR, 1.26; 95% CI, 1.11-1.43), and the ED visit occurring overnight (IRR, 1.93; 95% CI, 1.80-2.08).

**Table 3.** Association Between Patient Visit Characteristics and Behavioral Flag Presence in the Electronic Health Record

Covariate	IRR (95% CI)
Age, y	
16-24	1 [Reference]
25-34	1.41 (1.27-1.56)^a^
35-44	2.54 (2.16-3.00)^a^
45-54	1.61 (1.44-1.80)^a^
55-64	1.34 (1.19-1.51)^a^
≥65	0.43 (0.36-50)^a^
Black race	
Black	2.52 (2.31-2.76)^a^
White	1 [Reference]
Sex	
Female	1 [Reference]
Male	2.41 (2.19-2.65)^a^
Arrival by ambulance	1.26 (1.11-1.43)^a^
Emergency Severity Index	1.05 (1.00-1.11)
ED site	
Hospital 1 (tertiary referral center)	1 [Reference]
Hospital 2	1.02 (0.92-1.13)
Hospital 3 (level 1 trauma center)	0.47 (0.44-0.51)^a^
Weekend visit	1.14 (1.08-1.19)^a^
Shift	
Evening	1.09 (1.00-1.18)
Overnight	1.93 (1.80-2.08)^a^
Month	1.02 (1.01-1.03)
Year	1.50 (1.39-1.61)

Abbreviations: ED, emergency department; IRR, incidence rate ratio.

^a^ *P* < .001.

A total of 881 individuals met criteria for frequent ED utilization over the study period. These patients were flagged at higher rates (157 total individuals with flags, 178 flags per 1000 patients). Additionally, 500 patients presented to the ED with sickle cell–related chief concerns during the study period, accounting for 7223 visits, and 45 of these individuals had flags (9 flags per 1000 patients). eTable 3 in Supplement 1 provides subanalyses for these populations.

## Discussion

This cohort study analyzed ED visits across a large urban academic health system to investigate the presence of EHR behavioral flag notifications and differences between Black and White ED patients. This study has 3 main findings. First, behavioral flags were not widely used across a large cohort of patients presenting the ED. Second, when flags were used, there was a disparity across Black and White patients with a flag, with Black patients having a flag more often than White patients. Third, in this cohort, patients with a flag had less basic ED testing completed and longer treatment times and were more likely to be discharged. When excluding patients with sickle cell disease and those with frequent ED use, Black patients with a flag were received less ED clinical testing and treatment and were more likely to leave prior to full evaluation compared with White patients with a flag.

There is a developing body of research investigating the components of the EHR, structural racism, and clinical care. For example, a 2022 study^8^ found 2.54 greater odds of negative descriptor language within the EHR history and physical notes for hospitalized Black patients compared with White patients. A 2021 study^9^ observed more markers of clinician disbelief and judgment in the EHR of Black patients compared with White patients. Prior research has identified other racial and ethnic disparities in the provision of clinical care in the ED, including longer wait times and total lengths of stay, lower priority at triage, and higher death rates after and during ED visits.^10^ These unequal outcomes may have individual contributions, but can arise as easily and more persistently from differences in the structure of the care environment and architecture, including the EHR.^11,12^ Behavioral flags may represent another aspect of the EHR that could contribute to racial and ethnic disparities in outcomes and requires exploration. As these alerts become part of the EHR, it is important to continuously review the duration of the flag placement and appoint oversight to have flags reevaluated for content, validity, and duration.

There has been an increasing emphasis on well-being and safety for the health care workforce, which has been heightened by the COVID-19 pandemic. There remains a gap in understanding the intersection of these 2 important aspects of health care delivery.^13^ Behavioral flags have arisen to fill a gap in available interventions to address workplace violence, yet there is a paucity of research that investigates these flags and the potential for unintended consequences for equitable patient care. In this study, we note that flags were not commonly used across this ED setting (0.3% of patients had flags). Despite reports of high rates of violence against ED staff, these notifications were rarely in place. This may suggest a lack of reporting workplace violence or low perceived benefit in placing the flag. Understanding why and when flags are placed remains understudied in clinical environments, and future studies may explore who is placing flags, their reasoning or motivations, the effectiveness of flags in curtailing ED workplace violence, and the systems in place to oversee flag placement.

Although differences in ED clinical care, such as laboratory or radiology testing, were observed between Black and White patients once a flag was placed, these differences were small. This suggests that interventions aimed at reducing disparate care should first aim to debias the flag placement process, since they were disproportionately placed for Black patients. The aspects of clinical care used here (eg, number of tests ordered, time spent in room) are just a few aspects of clinical care and patient experience. Additional attention must also incorporate the role of sickle cell disease and the intersection of EHR flags, as in our subanalysis we observed increased disparities for individuals presenting to the ED with sickle cell disease–related concerns. Questions also arise from this study as to why Black patients were more likely to be flagged; the reasons for this, including structural factors and racial bias, require further study. This analysis demonstrated that flags were associated with care, which may in turn be associated with diagnostic error.

Our analysis found that disparities were present with regards to which patients were receiving flags, which represent an unintended outcome of a policy directed to improve workplace safety. Potential approaches to improve equity in care while keeping staff safe may include a systematic and team approach to review and authorize flag placement when an issue arises. Additionally, continuing review and consideration of removal of flags after subsequent visits by patients in which no further behavioral issues are noted. Patient flags may also benefit from a time limit, whereby the flag does not exist in the EHR indefinitely, but rather is removed after a defined duration of time (eg, 1 year from initial incident).

### Limitations

This study has some limitations. This was a retrospective cohort study conducted an academic, urban health system across 3 ED sites; thus, its findings may not generalize to other settings. Additionally, this study focused on ED EHR notes and flags and may not generalize to inpatient and outpatient settings with lower rates of violence or different clinical environments. The study also used self-reported race and ethnicity data within the EHR, which have been demonstrated in prior research to be of varying quality.^14^ The study used EHR data and did not include data on who was placing the flag or more granular insights on why the flag was placed, although this is a focus of future work. Outcomes recorded in EHR data may also not fully capture the patient experience influenced by placement of a behavioral flag, such as increased microaggressions from staff primed to think of a patient as disruptive. The study findings indicate observed differences in common ED clinical care metrics, such as length of stay or waiting time; however, how this may be associated with quality of care delivered remains unknown. In this study, we a priori excluded visits related to sickle cell disease, as this diagnosis predominately impacts Black patients, may be the cause of multiple ED visits, and may result in pain-related flags being placed within the context of ED care. Excluding sickle cell–related visits was necessary to enhance comparability and allow us to generalize results to all ED patients, but this analysis may understate the size of the racial disparity in flag placement.

## Conclusions

This cohort study found that behavioral flag notifications in the EHR were rarely used within ED visits. When they were used, there was a difference across race, with Black patients being more likely have a flag placed than White patients. We found that patients with a flag were less likely to be admitted and more likely to leave against medical advice, a difference that was amplified in Black patients. These findings suggest that behavioral flags are inequitably deployed in ED settings, replicating societal biases for subsequent waves of assumed criminality, from the racialization of the diagnoses of schizophrenia in the 1960s and 1970s, to the conceptualization of so-called *super predators* in the 1990s, to contemporary use of *agitated delirium* to justify violent police actions.^15^ Behavioral flags have important implications for patient care: they may introduce cognitive bias from clinicians providing acute care, since they often serve as alerts or advisory notifications when opening a patient’s EHR. Understanding when, and for whom, these flags are being implemented is important in how health systems think about providing equitable care while maintaining staff safety.

## Notes

Supplement 1. **eTable 1.** Patient- and Visit-Level Characteristics Comparing All Patients With and Without Behavioral Flags Excluding Sickle Cell Related Chief Concerns and Frequent Utilizing Patients **eTable 2.** Patient- and Visit-Level Characteristics Comparing Black and White Patients with Behavioral Flags Excluding Sickle Cell Related Chief Complaints and Frequent Utilizing Patients **eTable 3.** Patient- and Visit-Level Characteristics Comparing All Patients and High Utilizing Patients and Sickle Cell Related Concerns

Supplement 2.
Data Sharing Statement
