# Supplementary material for: Prevalence of Behavioral Flags in the Electronic Health Record Among Black and White Patients Visiting the Emergency Department
Source: JAMA Netw Open. 2023 Jan 19;6(1):e2251734. doi: 10.1001/jamanetworkopen.2022.51734 (PMC9857105; doi:10.1001/jamanetworkopen.2022.51734)
Supplement: Supplement 1. — eTable 1. Patient- and Visit-Level Characteristics Comparing All Patients With and Without Behavioral Flags Excluding Sickle Cell Related Chief Concerns and Frequent Utilizing Patients eTable 2. Patient- and Visit-Level Characteristics Comparing Black and White Patients with Behavioral Flags Excluding Sickle Cell Related Chief Complaints and Frequent Utilizing Patients eTable 3. Patient- and Visit-Level Characteristics Comparing All Patients and High Utilizing Patients and Sickle Cell Related Concerns [file jamanetwopen-e2251734-s001.pdf]

Supplemental Online Content

Agarwal AK, Seeburger E, O'Neill G, et al. Prevalence of behavioral flags in the electronic health record among Black and White patients visiting the emergency department. *JAMA Netw Open*. 2023;6(1):e2251734. doi:10.1001/jamanetworkopen.2022.51734

eTable 1. Patient- and Visit-Level Characteristics Comparing All Patients With and Without Behavioral Flags Excluding Sickle Cell Related Chief Concerns and Frequent Utilizing Patients

eTable 2. Patient- and Visit-Level Characteristics Comparing Black and White Patients with Behavioral Flags Excluding Sickle Cell Related Chief Complaints and Frequent Utilizing Patients

eTable 3. Patient- and Visit-Level Characteristics Comparing All Patients and High Utilizing Patients and Sickle Cell Related Concerns

This supplemental material has been provided by the authors to give readers additional information about their work.

© 2023 Agarwal AK et al. *JAMA Network Open.*

eTable 1. Patient- and Visit-Level Characteristics Comparing All Patients With and Without Behavioral Flags Excluding Sickle Cell Related Chief Concerns and Frequent Utilizing Patients

	Flag	No flag	
	**All Patients, N (%)**	**All Patients, N (%)**	**P Value**
Total Patients	522 (0.3%)	194,192 (99.7)	
Total Visits	1184 (0.3%)	382,658 (99.7)	
Age			P=.000
18-24	54 (10.3)	55,829 (14.6)	
25-34	132 (25.3)	81,953 (21.4)	
35-44	133 (25.5)	53,872 (14.2)	
45-54	100 (19.2)	53,230 (13.9)	
55-64	78 (14.9)	59,886 (15.7)	
65 and older	25 (4.8)	77,888 (20.4)	
Gender			P=.000
Male	289 (55.4)	157,900 (41.3)	
Female	233 (44.6)	224,756 (58.7)	
Race			P=.000
Black (Includes Hispanic/Latinx)	355 (68.0)	250,091 (65.4)	
White (includes Hispanic/Latinx)	167 (32.0)	132,567 (34.6)	
Insurance status			P=.000
Commercial	69 (5.8)	40,846 (10.7)	
Managed care	60 (5.1)	88,539 (23.1)	
Medicaid	750 (63.3)	132,206 (34.6)	
Medicare	240 (20.3)	98,452 (25.7)	
Self-pay	65 (5.5)	22,607 (5.9)	
Emergency Severity Index (ESI) Level			
ESI Level 1 (most acute)	7 (.6)	2,532 (.7)	P=.000
ESI Level 2	288 (25.0)	86,544 (23.2)	
ESI Level 3	595 (51.7)	193,311 (51.9)	
ESI Level 4	188 (16.4)	78,767 (21.1)	
ESI Level 5 (least acute)	53 (4.6)	9,511 (2.6)	
Psych	19 (1.7)	2,029 (.5)	
Emergency Department			
Hospital #1 (Tertiary Referral Center)	412 (34.8)	157,520 (41.1)	P=.000
Hospital #2	454 (38.3)	99,400 (26.0)	
Hospital #3 (Level 1 Trauma Center)	318 (26.9)	125,738 (32.9)	
Insurance status			
Commercial	69 (5.8)	40,846 (10.7)	P=.000
Managed care	60 (5.1)	88,539 (23.1)	
Medicaid	750 (63.3)	132,206 (34.6)	

© 2023 Agarwal AK et al. *JAMA Network Open.*

Medicare	240 (20.3)	98,452 (25.7)	
Self-pay	65 (5.5)	22,607 (5.9)	
	**Flag**	**No flag**	
	**Median (IQR)**	**Median (IQR)**	**P Value**
WR time (minutes)	20.27 (7.95-77.53)	29.63 (10.43-91.97)	P=.000
Wait to see clinician (minutes)	33.79 (15.87-82.75)	40.07 (18.72-95.60)	P=.000
In-room time (minutes)	222.95 (110.35-386.2)	224.75 (125.08-374.06)	p=.5965
ED LOS (minutes)	277.28 (149-461)	291 (173-466)	p=.0123
	**All Patients, N (%)**	**All Patients, N (%)**	**P Value**
Number of lab orders			
0 lab orders	415 (35.05)	104,287 (27.25)	P=.000
1-3 lab orders	133 (11.23)	46,539 (12.16)	
4 or more lab orders	636 (53.72)	231,832 (60.58)	
Number of medication orders			P=.000
0 medication orders	292 (24.66)	66,012 (17.55)	
1-3 medication orders	411 (34.71)	141,317 (36.93)	
4 or more medication orders	481 (40.62)	175,329 (45.82)	
Number of imaging orders			P=.000
None	616 (52.03)	152,086 (39.74)	
X-ray only	266 (22.47)	100,369 (26.23)	
Advanced imaging (CT, US, or MRI)	302 (25.51)	130,203 (34.03)	
Disposition			P=.000
Admit	177 (14.96)	69847 (18.16)	
Discharge	750 (63.4)	264911 (68.89)	
Left against medical advice or without being seen	200 (16.91)	26492 (6.89)	
Observation	23 (1.94)	16756 (4.36)	
Transfer	20 (1.69)	2951 (.77)	
Triaged	4 (.34)	957 (.25)	
Voided/Other	10 (.84)	2623 (.68)	
Urine Drug Screen (UDS) present	114 (21.84)	12,849 (6.60)	P=.000
Presence of intramuscular medication**	62 (11.87)	15,744 (8.09)	p=.001
Restraints used	1 (.19)	46 (.02)	p=.024
**Non-flag visits for patients who eventually received a behavioral flag during the study period were excluded **excludes tetanus vaccine administration*			

© 2023 Agarwal AK et al. *JAMA Network Open.*

eTable 2. Patient- and Visit-Level Characteristics Comparing Black and White Patients with Behavioral Flags Excluding Sickle Cell Related Chief Complaints and Frequent Utilizing Patients

	Black Patients, N (%)	White Patients, N (%)	P Value
# and percent with flag	355<br>(3.1 flags per 1,000 patients)	167<br>(2.0 flags per 1,000 patients)	p<.001
	**Black Pts with Flag**	**White Pts with Flag**	
	**Median (IQR)**	**Median (IQR)**	**P Value**
Waiting room time, minutes	20.7 (7.8-74.3)	15.9 (7.4-56.1)	p=.048
Wait to see clinician, minutes	33.7 (15.4-78.6)	29.4 (13.9-72.6)	p=.198
In-room time, minutes	224.9 (113.6-401.2)	255.6 (130.7-401.0)	p=.152
Length of stay, minutes	276.9 (144-476)	293.9 (160-439)	p=.883
	**N (%)**	**N (%)**	**P Value**
Number of lab orders			p=.11
0 lab orders	308 (36.9)	107 (30.6)	
1-3 lab orders	90 (10.8)	43 (12.3)	
4 or more lab orders	436 (52.3)	200 (57.1)	
Number of medication orders			p=.14
0 medication orders	219 (26.3)	73 (20.9)	
1-3 medication orders	283 (33.9)	128 (36.6)	
4 or more medication orders	332 (39.8)	149 (42.6)	
Number of imaging orders			p=.18
None	442 (53.0)	174 (49.7)	
X-ray only	192 (23.0)	74 (21.1)	
Advanced imaging (CT, US, or MRI)	200 (24.0)	102 (29.1)	
Disposition			p=.005
Admit	104 (12.5)	73 (20.8)	
Discharge	536 (64.4)	214 (61.1)	
Left against medical advice or without being seen	154 (18.5)	46 (13.1)	
Observation	15 (1.8)	8 (2.3)	
Transfer	14 (1.7)	6 (1.7)	
Triaged	3 (.4)	1 (.3)	
Voided/Other	8 (1.0)	2 (.6)	
Emergency Department			P=.000
Hospital of the University of Pennsylvania	306 (36.7)	106 (30.3)	
Pennsylvania Hospital	268 (32.1)	186 (53.1)	
Penn Presbyterian Medical Center	260 (31.2)	58 (16.6)	
Insurance status			p=.001
Commercial	43 (5.2)	26 (7.4)	
Managed care	29 (3.5)	31 (8.9)	

Medicaid	548 (65.7)	202 (57.7)	
Medicare	166 (19.9)	74 (21.1)	
Self-pay	48 (5.8)	17 (4.9)	
Emergency Severity Index (ESI) Level			p=.07
ESI Level 1 (most acute)	3 (.4)	4 (1.1)	
ESI Level 2	202 (24.2)	86 (24.6)	
ESI Level 3	407 (48.8)	188 (53.7)	
ESI Level 4	142 (17.0)	46 (13.1)	
ESI Level 5 (least acute)	44 (5.3)	9 (2.6)	
Psych	11 (1.3)	8 (2.3)	
Unknown	25 (3.0)	9 (2.6)	

© 2023 Agarwal AK et al. *JAMA Network Open.*

eTable 3. Patient- and Visit-Level Characteristics Comparing All Patients and High Utilizing Patients and Sickle Cell Related Concerns

Patient Characteristics Comparing All Comers and High Utilizers				
	All Comers	All Comers (Excluding Many Visitors)	Many Visitors (>18 visits)	
	**All Patients, N (%)**	**All Patients, N (%)**	**All Patients, N (%)**	**P Value**
Total Patients	195,601	194,719	881	
Total Visits	420,007	385,773	34,234	
Age				p<.01
18-24	31,497 (16.1)	31,431 (16.14)	66 (7.49)	
25-34	43,577 (22.28)	43,382 (22.28)	195 (22.13)	
35-44	28,397 (14.52)	28,231 (14.5)	166 (18.84)	
45-54	26,714 (13.66)	26,540 (13.63)	174 (19.75)	
55-64	28,818 (14.73)	28,663 (14.72)	155 (17.59)	
65 and older	36,598 (18.71)	36,473 (18.73)	125 (14.19)	
Gender				p<.01
Female	110,890 (56.69)	110,436 (56.72)	454 (51.53)	
Male	84,710 (43.31)	84,283 (43.28)	427 (48.47)	
Race				p<.01
Black (Includes Hispanic/Latinx)	113,638 (58.1)	112,908 (57.98)	730 (82.86)	
White (includes Hispanic/Latinx)	81,963 (41.9)	81,812 (42.02)	151 (17.14)	
**Patient Characteristics Comparing All Comers and Chief Complaint Related to Sickle Cell**				
	All Comers	All Comers, Excluding Sickle Cell Chief Complaint	Sickle Cell Chief Complaint	
	**All Patients, N (%)**	**All Patients, N (%)**	**All Patients, N (%)**	**P Value**
Total Patients	195,601	195,101	500	
Total Visits	420,007	412,784	7,223	
Age				p<.01
18-24	31,497 (16.1)	31,358 (16.1)	139 (27.8)	
25-34	43,577 (22.28)	43,361 (22.22)	216 (43.20)	
35-44	28,397 (14.52)	28,312 (14.51)	85 (17)	
45-54	26,714 (13.66)	26,677 (13.67)	37 (7.4)	
55-64	28,818 (14.73)	28,800 (14.76)	18 (3.6)	
65 and older	36,598 (18.71)	36,593 (18.76)	5 (1)	
Gender				p=.63
Female	110,890 (56.69)	110,607 (56.7)	283 (56.6)	
Male	84,710 (43.31)	84,493 (43.3)	217 (43.4)	
Race				p<.01
Black (Includes Hispanic/Latinx)	113,638 (58.1)	113,145 (58)	493 (98.6)	
White (includes Hispanic/Latinx)	81,963 (41.9)	81,956 (42)	7 (1.4)	

© 2023 Agarwal AK et al. *JAMA Network Open.*

Visit-Level Characteristics Comparing All Comers and High Utilizers				
	All Comers (N, %)	All Comers Excluding Many Visitors (N, %)	Many Visitors (N, %)	P Value
# and percent with flag	683 (3.5 flags per 1,000 patients)	526 (2.7 flags per 1,000 patients)	157 (178 flags per 1,000 patients)	p<.01
	**Median (IQR)**	**Median (IQR)**	**Median (IQR)**	**P Value**
Waiting room time, minutes	29.23 (10.3-91.77)	29.55 (10.42-91.92)	25.63 (9.47-89.53)	p<.01
Wait to see clinician, minutes	40.03 (18.67-96)	40.05 (18.7-95.6)	40 (18.25-101.4)	p=.09
In-room time, minutes	226.2 (125.28-376.49)	225.37 (125.42-374.98)	235.43 (123.25-394.08)	p<.01
Length of stay, minutes	292 (172-467)	292 (173-466.01)	292 (160.22-477)	p<.01
	**N (%)**	**N (%)**	**N (%)**	**P Value**
Number of lab orders				
0 lab orders	117,051 (27.87)	105,302 (27.30)	11,749 (34.32)	p<.01
1-3 lab orders	50,774 (12.09)	46,930 (12.17)	3,844 (11.23)	
4 or more lab orders	252,182 (60.04)	233,541 (60.54)	18,641 (54.45)	
Number of medication orders				
0 medication orders	74,393 (17.71)	66,767 (17.31)	7,626 (22.28)	p<.01
1-3 medication orders	153,400 (36.52)	142,370 (36.91)	11,030 (32.22)	
4 or more medication orders	192,214 (45.76)	176,636 (45.79)	15,578 (45.5)	
Number of imaging orders				
None	172,939 (41.18)	153,675 (39.85)	19,264 (56.27)	p<.01
X-ray only	110,308 (26.27)	101,554 (26.33)	8,754 (25.57)	
Advanced imaging (CT, US, or MRI)	136,662 (32.55)	130,448 (33.82)	6,214 (18.15)	
Disposition				
Admit	76,054 (18.11)	70,037 (18.15)	6,017 (17.58)	p<.01
Discharge	288,439 (68.67)	265,694 (68.87)	22,745 (66.44)	
Left against medical advice or without being seen	30,287 (7.21)	26,697 (6.92)	3,590 (10.49)	
Observation	18,052 (4.3)	16,780 (4.35)	1,272 (3.72)	
Transfer	3,157 (.75)	2,971 (.77)	186 (.54)	
Triaged	1,029 (.24)	961 (.25)	68 (.20)	
Voided/Other	2,989 (.71)	2,633 (.68)	356 (1.04)	
Emergency Department				
Hopsital of the University of Pennsylvania	171,447 (40.82)	158,554 (41.1)	12,893 (37.66)	p<.01
Pennsylvania Hospital	112,009 (26.67)	100,529 (26.06)	11,480 (33.53)	
Penn Presbyterian Medical Center	136,551 (32.51)	126,690 (32.84)	9,861 (28.8)	
Insurance status				
Commercial	42,505 (10.12)	41,039 (10.64)	1,462 (4.27)	p<.01
Managed care	90,477 (21.54)	88,730 (23)	1,747 (5.1)	
Medicaid	154,918 (36.89)	134,112 (34.77)	20,806 (60.78)	
Medicare	108,912 (25.93)	99,125 (25.7)	9,787 (28.59)	
Self-pay	23,190 (5.52)	22,759 (5.9)	431 (1.26)	
Emergency Severity Index (ESI) Level				
ESI Level 1 (most acute)	2,626 (.64)	2,545 (.68)	81 (.24)	p<.01
ESI Level 2	94,185 (23.03)	87,300 (23.24)	6,885 (20.66)	
ESI Level 3	212,692 (52)	194,884 (51.87)	17,808 (53.43)	
ESI Level 4	84,437 (20.64)	79,236 (21.09)	5,201 (15.6)	
ESI Level 5 (least acute)	12,653 (3.09)	9,650 (2.57)	3,003 (9.01)	
Psych	2,445 (.6)	2,092 (.56)	353 (1.06)	

© 2023 Agarwal AK et al. *JAMA Network Open.*

Visit-Level Characteristics Comparing All Comers versus Chief Complaint of Sickle Cell				
	**All Comers, N (%)**	**All Comers Excluding Sickle Cell, N (%)**	**Chief Complaint of Sickle Cell, N (%)**	**P Value**
# and percent with flag	683 (3.5 flags per 1,000 patients)	638 (3.3 flags per 1,000 patients)	45 (90 flags per 1,000 patients)	p<.01
	**Median (IQR)**	**Median (IQR)**	**Median (IQR)**	**P Value**
Waiting room time, minutes	29.23 (10.33-91.77)	29.38 (10.35-92.08)	21.97 (9.75-76.32)	p<.01
Wait to see clinician, minutes	40.03 (18.67-96)	40.1 (18.7-96.12)	36.88 (17.18-89.18)	p<.01
In-room time, minutes	226.2 (125.28-376.49)	224.75 (123.95-375.68)	283.67 (200.07-408.35)	p<.01
Length of stay, minutes	292 (172-467)	291 (171-467)	330 (228.67-490)	p<.01
	**N (%)**	**N (%)**	**N (%)**	**P Value**
Number of lab orders				p<.01
0 lab orders	117,051 (27.87)	116,276 (28.17)	775 (10.73)	
1-3 lab orders	50,774 (12.09)	50,339 (12.19)	435 (6.02)	
4 or more lab orders	252,182 (60.04)	246,169 (59.64)	6,013 (83.25)	
Number of medication orders				p<.01
0 medication orders	74,393 (17.71)	74,171 (17.97)	222 (3.07)	
1-3 medication orders	153,400 (36.52)	152,614 (36.97)	786 (10.88)	
4 or more medication orders	192,214 (45.76)	185,999 (45.06)	6,215 (86.04)	
Number of imaging orders				p<.01
None	172,939 (41.18)	169,327 (41.03)	3,612 (50.01)	
X-ray only	110,308 (26.27)	107,351 (26.01)	2,957 (40.94)	
Advanced imaging (CT, US, or MRI)	136,662 (32.55)	136,008 (32.96)	654 (9.05)	
Disposition				p<.01
Admit	76.054 (18.11)	72,786 (17.63)	3,268 (45.24)	
Discharge	288,439 (68.67)	284,842 (69.01)	3,597 (49.8)	
Left against medical advice or without being seen	30,287 (7.21)	29,996 (7.27)	291 (4.03)	
Observation	18,052 (4.3)	18,016 (4.36)	36 (.5)	
Transfer	3,157 (.75)	3,141 (.76)	16 (.22)	
Triaged	1,029 (.24)	1,023 (.25)	6 (.08)	
Voided/Other	2,989 (.71)	2,980 (.72)	9 (.12)	
Emergency Department				p<.01
Hopsital of the University of Pennsylvania	171,447 (40.82)	169,743 (41.12)	1,704 (23.59)	
Pennsylvania Hospital	112,009 (26.67)	107,628 (26.07)	4,381 (60.65)	
Penn Presbyterian Medical Center	136,551 (32.51)	135,413 (32.8)	1,138 (15.76)	
Insurance status				p<.01
Commercial	42,505 (10.12)	42,160 (10.21)	342 (4.73)	
Managed care	90,477 (21.54)	89,538 (21.69)	939 (13)	
Medicaid	154,918 (36.89)	150,526 (36.47)	4,392 (60.81)	
Medicare	108,912 (25.93)	107,442 (26.03)	1,470 (20.35)	
Self-pay	23,190 (5.52)	23,110 (5.6)	80 (1.11)	
Emergency Severity Index (ESI) Level				p<.01
ESI Level 1 (most acute)	2,626 (.64)	2,626 (.65)	0 (0)	
ESI Level 2	94,185 (23.03)	92,871 (23.11)	1,314 (18.2)	
ESI Level 3	212,692 (52)	206,797 (51.47)	5,895 (81.65)	
ESI Level 4	84,437 (20.64)	84,427 (21.01)	10 (.14)	
ESI Level 5 (least acute)	12,653 (3.09)	12,652 (3.15)	1 (.01)	
Psych	2,445 (.6)	2,445 (.61)	0 (0)	

© 2023 Agarwal AK et al. *JAMA Network Open.*
