# Supplementary material for: Prevalence of Behavioral Flags in the Electronic Health Record Among Black and White Patients Visiting the Emergency Department
Source: JAMA Netw Open. 2023 Jan 19;6(1):e2251734. doi: 10.1001/jamanetworkopen.2022.51734 (PMC9857105; doi:10.1001/jamanetworkopen.2022.51734)
Supplement: Supplement 2. — Data Sharing Statement [file jamanetwopen-e2251734-s002.pdf]

Data Sharing Statement

Agarwal. Prevalence of Behavioral Flags in the Electronic Health Record Among Black and White Patients Visiting the Emergency Department. *JAMA Netw Open*. Published January 19, 2023. doi:10.1001/jamanetworkopen.2022.51734

Data
Data available: Yes
Data types: Deidentified participant data
How to access data: data will be sent with approval, available from corresponding author anish.agarwal@pennmedicine.upenn.edu
When available: With publication

Supporting Documents
Document types: None

Additional Information
Who can access the data: researchers whose proposed use of the data has been approved
Types of analyses: for any purpose
Mechanisms of data availability: after approval of a proposal
